# Supplementary material for: The transcriptional regulator ZNF398 mediates pluripotency and epithelial character downstream of TGF-beta in human PSCs
Source: Nat Commun. 2020 May 12;11:2364. doi: 10.1038/s41467-020-16205-9 (PMC7217929; doi:10.1038/s41467-020-16205-9)
Supplement: Supplementary file 1 — Supplementary Information [file 41467_2020_16205_MOESM1_ESM.pdf]

Supplementary Information

The transcriptional regulator ZNF398 mediates pluripotency and epithelial character downstream of TGF-beta in human PSCs

Zorzan et al.

Supplementary Fig. 1

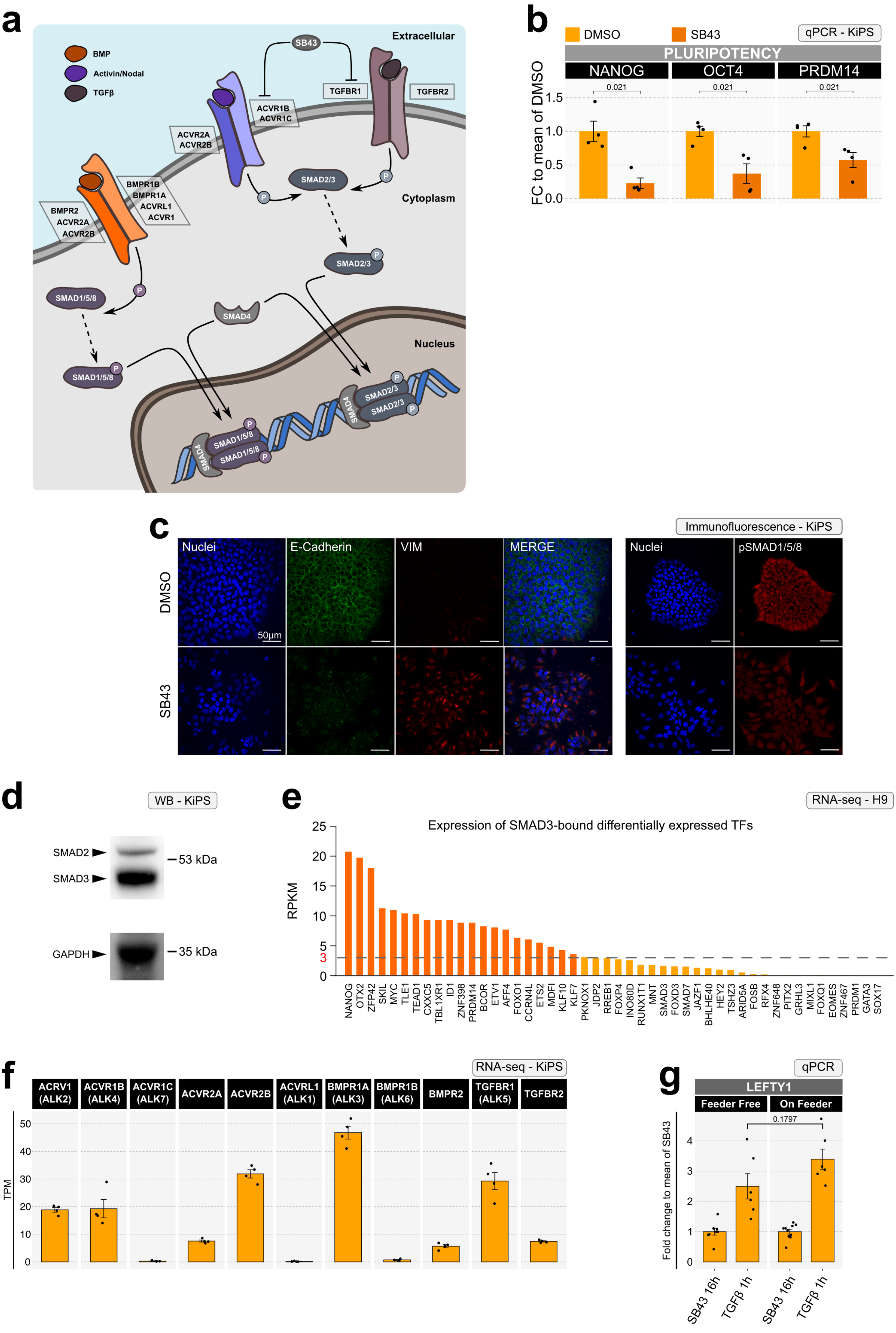

Supplementary Fig. 1: Molecular characterization of hPSCs.

a Diagram representing the ligands, the receptors and the SMAD proteins of the TGF-beta superfamily. **b** Gene-expression analysis by qPCR of KiPS treated with DMSO or SB43 for 5 days. Bars indicate the mean ± SEM of four independent experiments, shown as dots. Expression was normalised to the mean of DMSO samples. Unpaired two-tailed Mann–Whitney *U* test. Source data are provided as a Source Data file. **c** Left: Immunostaining for E-Cadherin (CDH1) shows a reduction of expression after 5 days of SB43 treatment, while immunostaining for VIMENTIN (VIM) shows an increased expression. Righ: Immunostaining for pSMAD1/5/8 shows a reduction of expression after 5 days of SB43 treatment. As a negative control, we checked if the BMP pathway was affected after 5 days of SB43 treatment. Representative images of three independent experiments are shown. See also Fig. 1a for immunostaining for pSMAD3 and for pluripotency markers. **d** Western Blot of KiPS. The antibody used recognizes both SMAD2 and SMAD3 proteins. Note that SMAD3 is much more abundant than SMAD2. GAPDH was used as loading control. Representative of two independent experiments. Uncropped gels are provided as a Source Data file. **e** Bar plot showing RNA-seq data of H9 (WA09) (data from[1], GSE24447, GSM602289). Bars indicate absolute expression (RPKM) of SMAD3-bound and differentially expressed Transcription Factors (TFs). Dashed line indicates the chosen threshold (>3 RPKM) and dark orange bars highlight the TFs above it. See also Fig. 1c. **f** Barcharts showing gene expression analysis by RNA-seq of KiPS. The receptors mediating signaling of TGF-beta (TGFBR1 and TGFBR2) and of Nodal/Activin (ACVR1B, ACVR2A, ACVR2B) are robustly expressed. Bars indicate the mean ± SEM of four independent experiments, shown as dots. Absolute expression is reported as TPM. Source data are provided as a Source Data file. **g** Gene expression analysis by qPCR of LEFTY1 after TGF-beta induction. Cells were treated overnight (16h) with SB43 and stimulated with TGF-beta for 1h. Induction of LEFTY1 was comparable in the presence or absence of feeders. Bars indicate mean ± SEM of n= 8, 6, 10, 6 biological replicates over six independent experiments shown as dots in Feeder-free and On Feeders condition, respectively. Expression was normalised to the mean of SB43 samples. Unpaired two-tailed *t*-test. See also Fig. 2a. Source data are provided as a Source Data file.

Supplementary Fig. 2

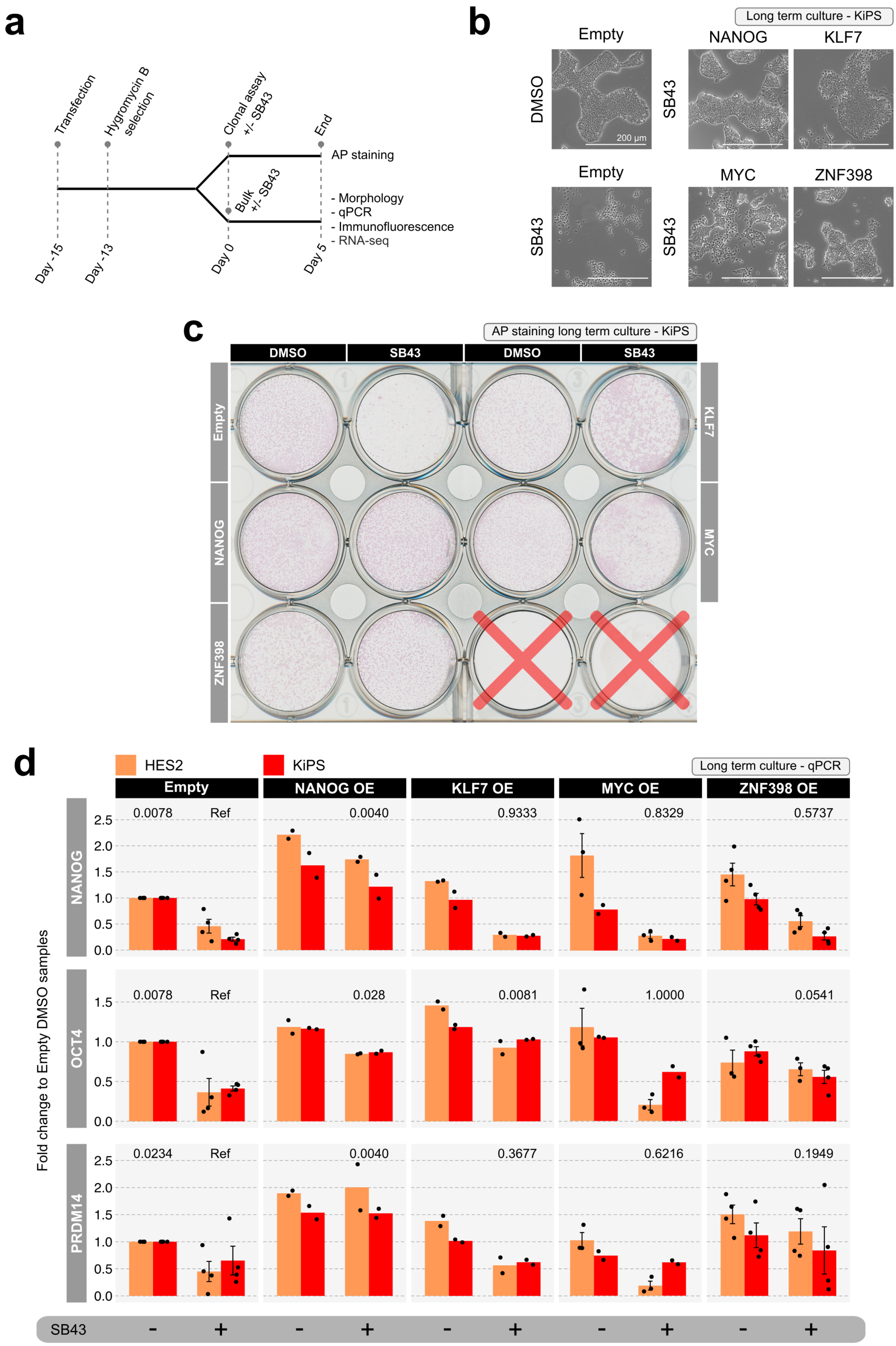

Supplementary Fig. 2: Experimental approach for identification of pluripotency regulators in hPSCs.

a Experimental approach used to test the functional role of SMAD3 targets identified in Fig. 1 and 2. hPSCs were co-transfected with pBase helper plasmid (encoding for the transposase) and a piggyBac vector allowing genomic integration and the stable expression of candidate SMAD3 targets or an empty vector (Empty). After 48 hours, hygromycin selection (200 µg/ml) was added for at least 2 weeks. After selection, cells were cultured with or without SB43 for 5 days and analysed as indicated. **b** Morphology of KiPS colonies stably expressing empty vector (Empty) in presence of DMSO or SB43 and KiPS stably expressing NANOG, KLF7, MYC or ZNF398 in presence of SB43 for 4 passages. Representative images of three independent experiments are shown. **c** AP staining of KiPS colonies stably expressing empty vector (Empty) in presence of DMSO or SB43 and KiPS stably expressing NANOG, KLF7, MYC or ZNF398 in presence of SB43 for 4 passages. Representative of two independent experiments. **d** Gene expression analysis by qPCR of HES2 (orange bars) and KiPS (red bars) stably expressing an Empty vector or the NANOG, KLF7, MYC and ZNF398 treated with or without SB43 for more of 3 passages. Bars indicate mean ± SEM of independent experiments, shown as dots (n=4, 4 for Empty OE; n=2, 2 for NANOG overexpression; n=2, 2 for KLF7 overexpression; n=3, 2 for MYC overexpression; n=4, 4 for ZNF398 in HES2 and KiPS, respectively). Expression was normalised to the Empty DMSO samples. Unpaired two-tailed Mann–Whitney *U* test, using the Empty vector SB43 sample as reference. Source data are provided as a Source Data file.

Supplementary Fig. 3

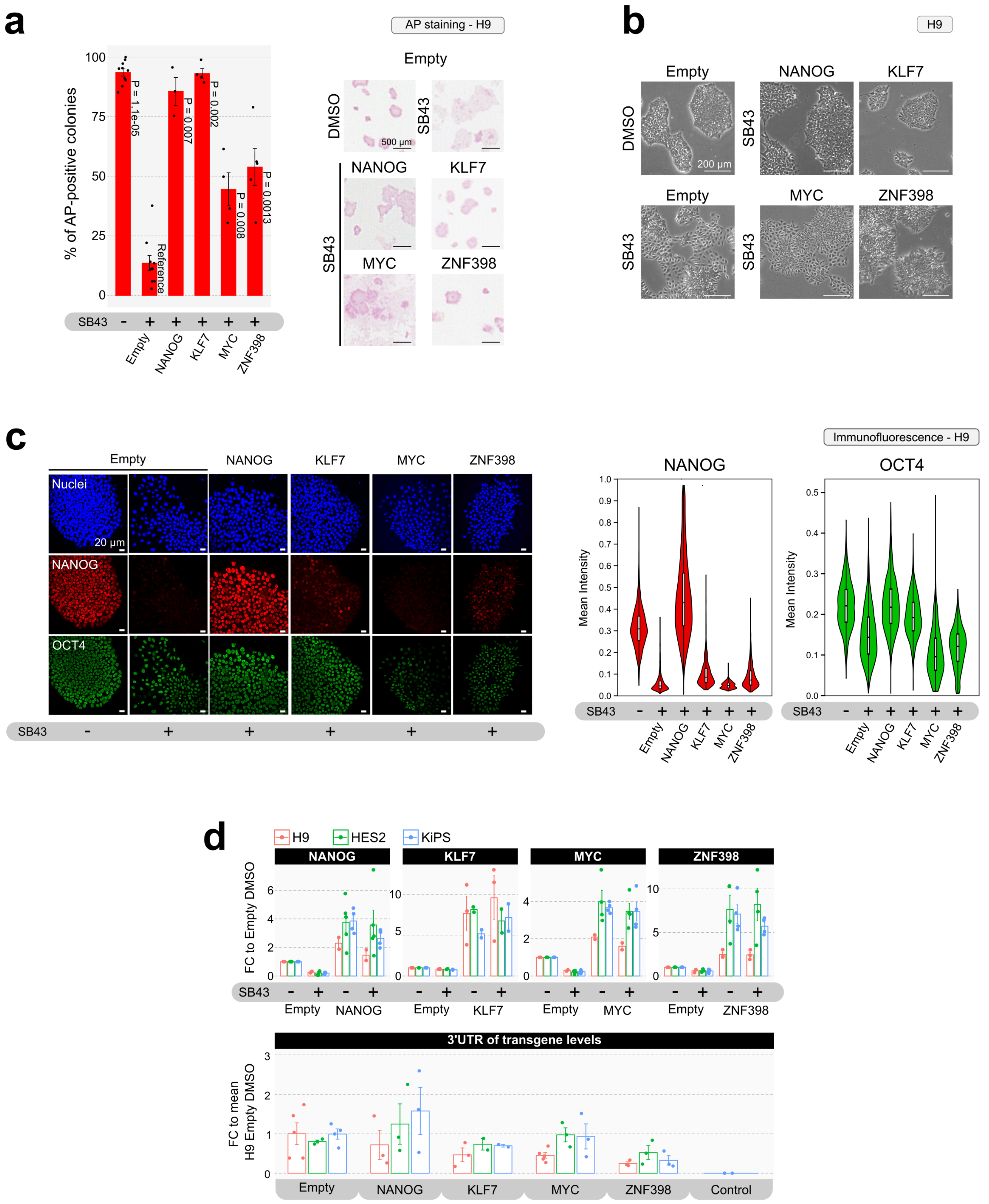

Supplementary Fig. 3: Functional identification of pluripotency regulators in H9 cells.

a Left: Clonal assay quantification of H9 stably expressing an empty vector, NANOG, KLF7, MYC or ZNF398. Five thousand cells were seeded at clonal density in the presence of DMSO or SB43 (2.5 μM) and stained for alkaline phosphatase (AP) after 5 days. Bars indicate mean ± SEM percentage of AP positive colonies. Independent experiments are shown as dots (n=10, 10 for Empty in DMSO and SB43, respectively; n=3 for NANOG SB43; n=4 for KLF7 SB43; n=4 for MYC SB43; n=5 for ZNF398 SB43). Unpaired two-tailed Mann–Whitney *U* test. Right: Representative AP staining images of clonal assay performed with H9. Source data are provided as a Source Data file. **b** Morphology of H9 colonies stably expressing empty vector (Empty) in presence of DMSO or SB43 (2.5 μM) and H9 stably expressing NANOG, KLF7, MYC or ZNF398 in presence of SB43 for 5 days. Representative images of three independent experiments are shown. **c** Left: Immunostaining for the pluripotency markers NANOG and POU5F1/OCT4 of H9 transfected with an empty vector (Empty) in presence of DMSO or SB43 (2.5 μM) and H9 stably expressing NANOG, KLF7, MYC or ZNF398 in presence of SB43 for 5 days. Representative images of three independent experiments are shown. Right: Violin plots showing fluorescence intensity quantification of NANOG and OCT4. For each condition, at least 800 nuclei from 5 random fields were analysed. Box plot indicates 25th, 50th and 75th percentile; whiskers indicate minimum and maximum. Source data are provided as a Source Data file. **d** Top: evaluation of overexpression levels by qPCR for H9, HES2 and KiPS (red, green and blue bars respectively) stably expressing an empty vector, NANOG, KLF7, MYC or ZNF398 and treated with SB43 or DMSO for 5 days. Bars indicate mean ± SEM of independent experiments shown as dots (n=2, 5, 4 for NANOG; n=3, 2, 2 for KLF7; n=2, 4, 4 for MYC; n=3, 4, 4 for ZNF398 in H9, HES2 and KiPS, respectively). Expression was normalised to Empty DMSO sample. We observe consistently reduced transgene expression for MYC and ZNF398 in H9 cells which might explain the reduced phenotypic effect. Bottom: Measurement of transgene expression levels by qPCR. Primers were designed to recognise a portion of the 3'UTR shared by all transgenes, allowing direct comparison among different vectors and specific detection of exogenous transcripts. Data for H9, HES2 and KiPS (red, green and blue bars respectively) stably expressing an empty vector, NANOG, KLF7, MYC or ZNF398 and treated with DMSO for 5 days are shown. Bars indicate mean ± SEM of independent experiments shown as dots (n=5, 3, 4 for Empty; n=3, 3, 3 for NANOG; n=3, 2, 2 or KLF7; n=5, 3, 3 for MYC; n=3, 3, 3 for ZNF398 in H9, HES2 and KiPS, respectively). Expression was normalised to H9 Empty samples. Untransfected KiPS samples serve as negative control. Exogenous expression was comparable among the 4 candidates, however it was consistently lower in H9 cells, especially for MYC and ZNF398. Source data are provided as a Source Data file.

Supplementary Fig. 4

a

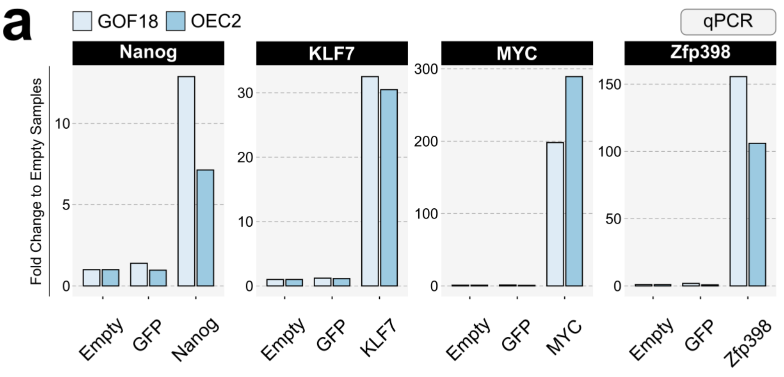

b

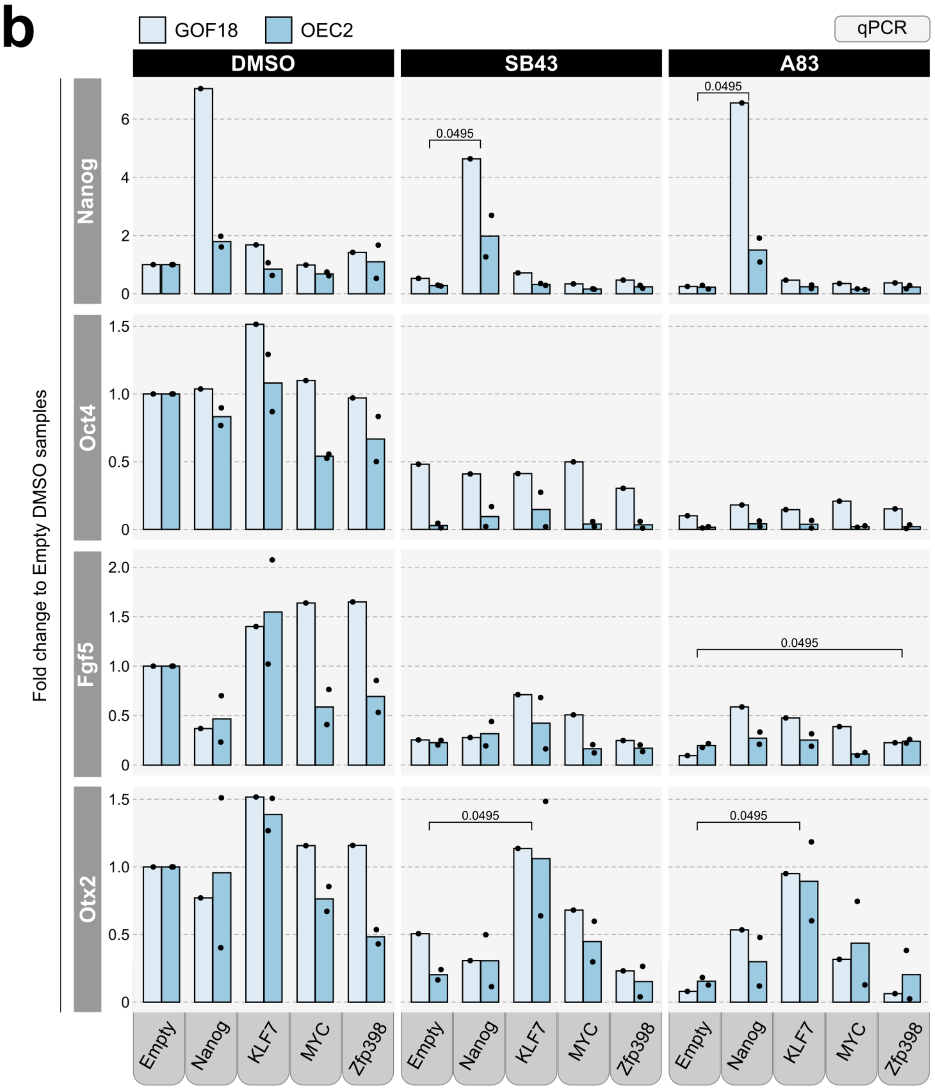

Supplementary Fig. 4: Functional identification of pluripotency regulators in mEpiSCs.

a Evaluation of overexpression levels by qPCR of GOF18 and OEC2 (light and dark blue bars respectively) stably expressing an empty vector, GFP (as a further control), Nanog, KLF7, MYC or Zfp398. Expression was normalised to Empty DMSO samples. A representative experiment for each line is shown. Source data are provided as a Source Data file. **b** Gene expression analysis by qPCR of GOF18 and OEC2 (light and dark blue bars respectively) stably expressing an empty vector, Nanog, KLF7, MYC or Zfp398 treated with DMSO, SB43 (1 µM) or A83-01 (A83, 1µM) for 2 days. A83 is a second inhibitor of TGFBR1 and ACVR1b/c. Bars indicate mean ± SEM of independent experiments, shown as dots (n=1 in GOF18 and n=2 in OEC2 for all candidates). Expression was normalised to the Empty DMSO samples. Unpaired two-tailed Mann–Whitney *U* test. Source data are provided as a Source Data file.

Supplementary Fig. 5

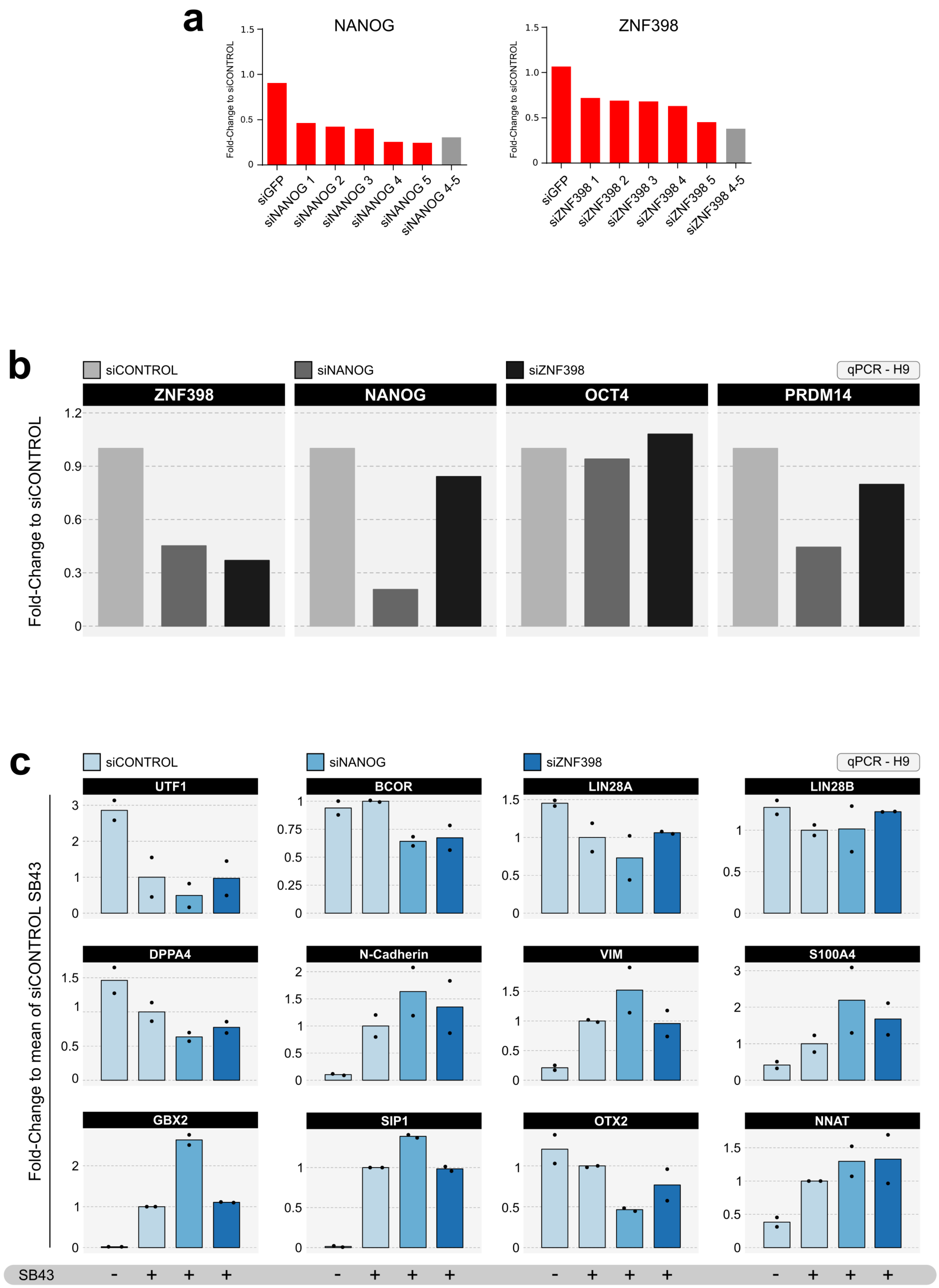

Supplementary Fig. 5: siRNA validation.

a Validation of the siRNAs used in this work. H9 were transfected with the indicated siRNA and analysed after 3 days by qPCR for the indicated genes (NANOG or ZNF398). The siGFP was used as a further control. For each gene, 5 independent siRNAs have been tested and the 2 showing the best knockdown efficiency have been used as pool (grey bars). A representative experiment is shown. Source data are provided as a Source Data file. **b** Gene expression analysis by qPCR of H9 transfected with the indicated siRNA (siCONTROL, siNANOG pool or siZNF398 pool) for 5 days. A representative experiment is shown. Expression was normalised to the siCONTROL. See also Fig. 5d. Source data are provided as a Source Data file. **c** Gene expression analysis by qPCR of H9 transfected with the indicated siRNA (siCONTROL, siNANOG pool or siZNF398 pool) with or without SB43 for 5 days. Bars indicate the mean of two independent experiments shown as dots. Expression was normalised to the mean of siCONTROL in SB43 samples. See also Fig. 5d. Source data are provided as a Source Data file.

Supplementary Fig. 6

a

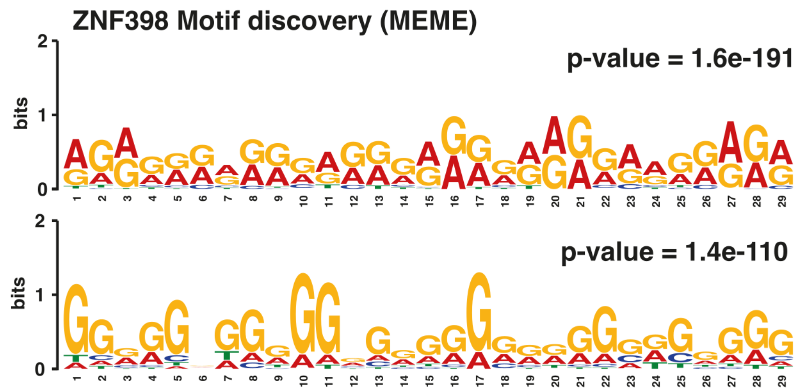

b

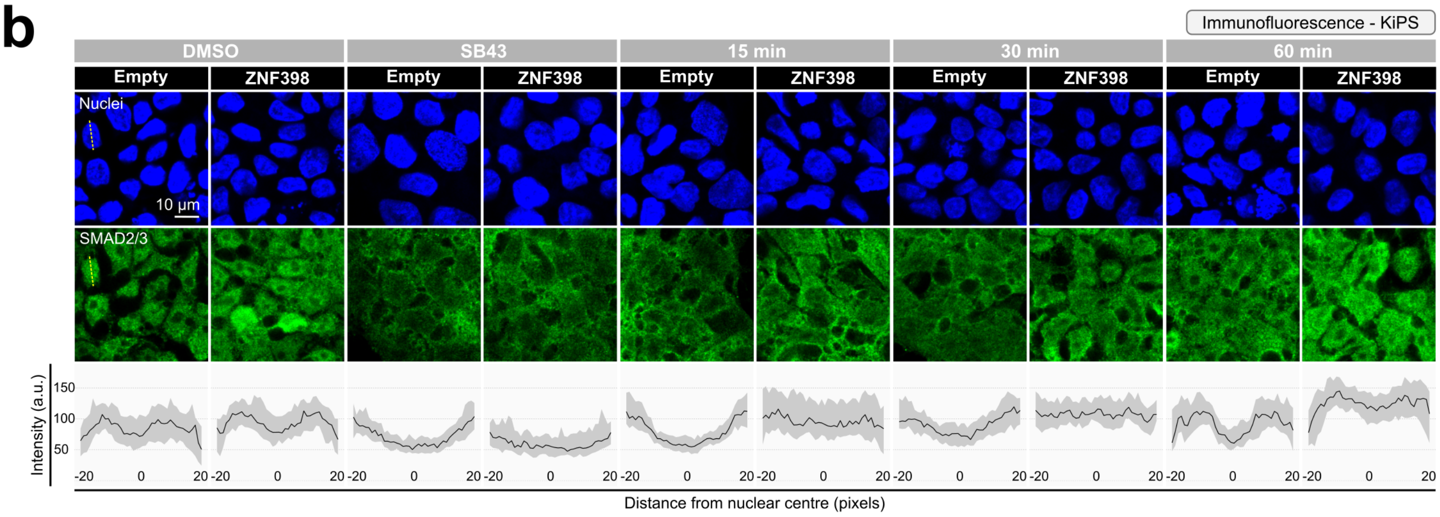

c

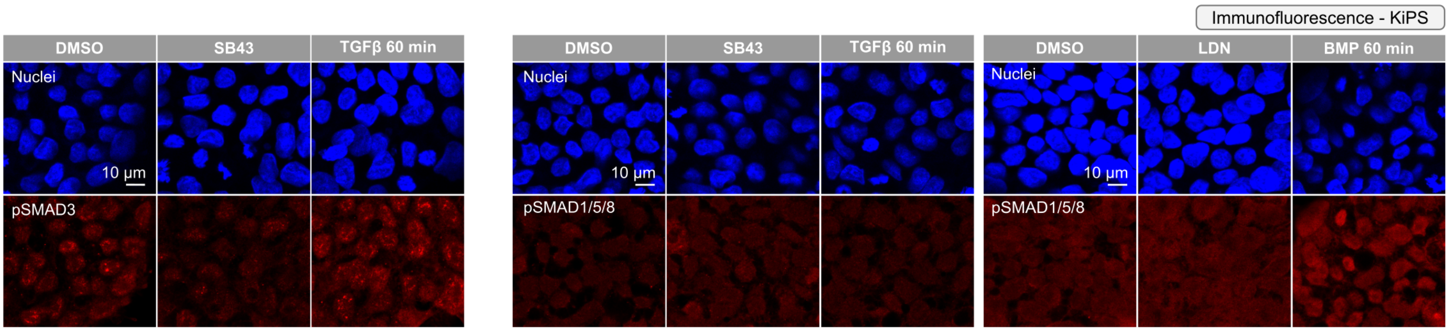

d

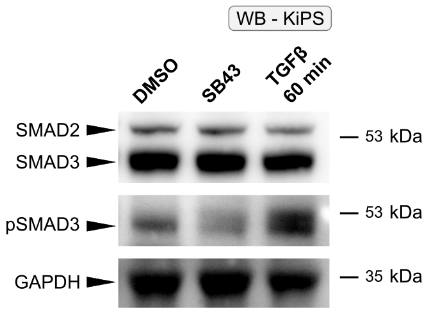

Supplementary Fig. 6: Effect of ZNF398 on R-SMADs.

a DNA binding motifs associated with ZNF398 peaks. P-value was calculated using MEME (v4.10.1)[2]. **b** Top: Immunostaining for SMAD2/3 of KiPS stably expressing an empty vector (Empty) control or ZNF398 treated with DMSO, SB43 for 16 hours, or after TGF-beta induction (2ng/ml) for 15, 30 and 60 minutes (min). Representative images of two independent experiments are shown. Bottom: line charts showing SMAD2/3 intensity in arbitrary units across hPSCs (dashed yellow line). On X axis, distance is expressed in pixels where 0 is the estimated nuclear centre and +/-20 correspond to the cytoplasm. For each condition, 48 cells from 6 randomly selected fields were analysed to calculate SMAD2/3 median intensity and 1st-3rd quartile interval, respectively represented by the black line and the shaded area. U-shaped trend indicates that SMAD2/3 is mainly cytoplasmic (Empty-SB43), conversely when SMAD2/3 is mostly nuclear the trend is flipped (ZNF398-60 min). Flat trend suggests that SMAD2/3 is equally distributed between cytoplasm and nucleus (ZNF398-15 min). Source data are provided as a Source Data file. **c** Left: Immunostaining for pSMAD3 of KiPS treated with DMSO, SB43 for 16 hours, or after TGF-beta induction (2ng/ml) for 60 min. Center: Representative images of immunostaining for pSMAD1/5/8 of KiPS treated with DMSO, SB43 for 16 hours, or after TGF-beta induction (2ng/ml) for 60 min. Note that the level of pSMAD1/5/8 is unchanged upon SB43 or TGF-beta induction treatment. Right: Immunostaining for pSMAD1/5/8 of KiPS treated with DMSO, 0.1 µM of LDN (LDN193189, inhibitor of BMP types I receptors ALK2 (ACRV1), ALK3 (BMPR1A), ALK6 (BMPR1B)) for 16 hours, or after BMP induction (100 ng/ml) for 60 min. As a positive control, we checked if upon BMP induction, the pSMAD1/5/8 were upregulated. Representative images of three independent experiments are shown. **d** Western Blot for SMAD2/3 and pSMAD3 of KiPS treated with DMSO, SB43 for 16 hours, or after TGF-beta induction (2 ng/ml) for 60 min. GAPDH was used as loading control. Note that the level of pSMAD3 is upregulated upon TGF-beta induction, while total SMAD2/3 are unchanged. Representative of two independent experiments. Uncropped gels are provided as a Source Data file.

Supplementary Fig. 7

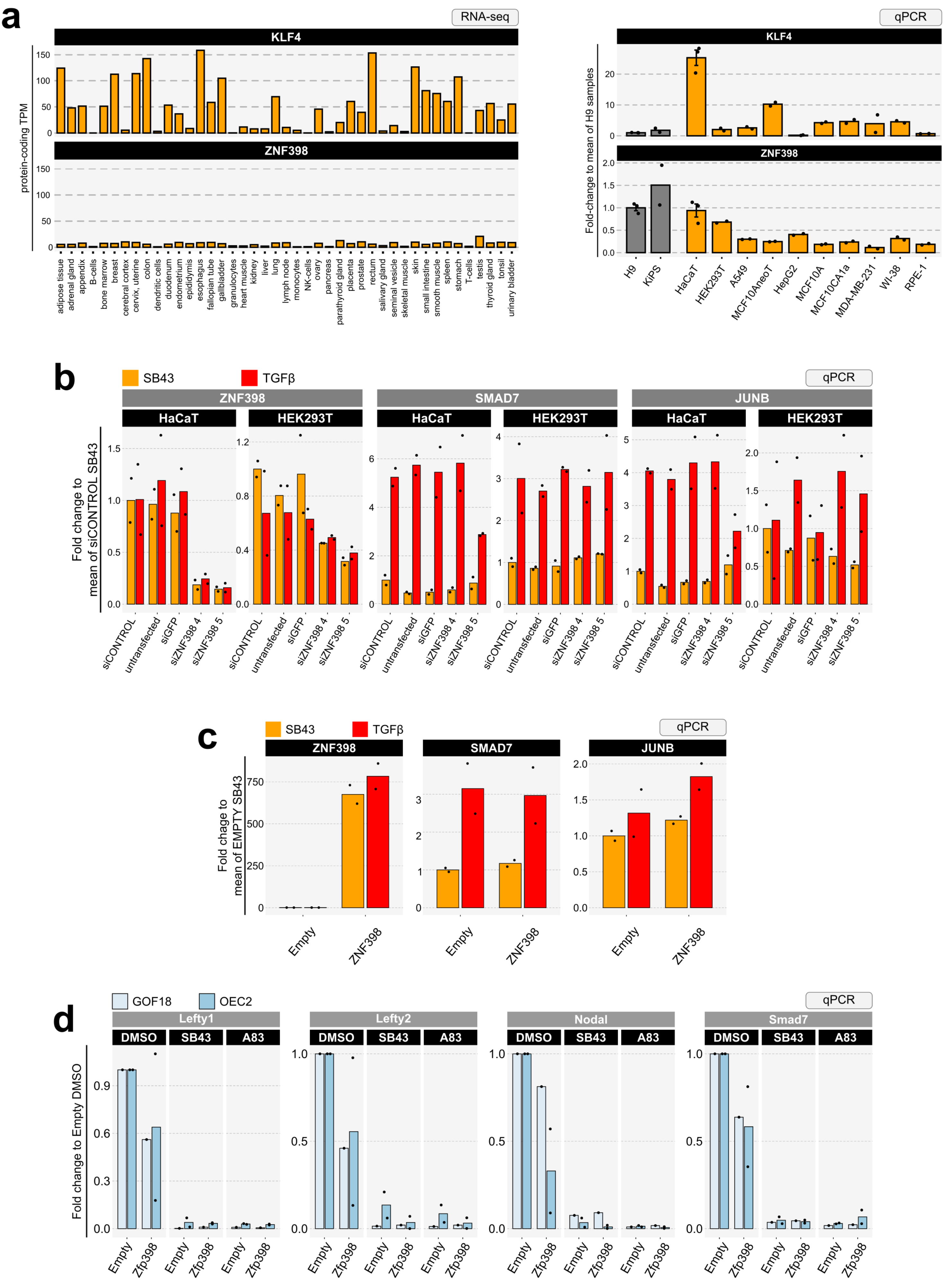

Supplementary Fig. 7: The role of ZNF398 as an activator of TGF-beta signal is hPSC-specific.

a Left: Analysis of RNA-seq data from HPA (Human Protein Atlas, available from[3]) of the indicated healthy tissues. Absolute expression is reported as protein-coding TPM. Note that ZNF398 is expressed at very low levels in all these healthy tissues. As a comparison, the pluripotency factor KLF4 shows robust expression in multiple tissues. Right: Gene expression analysis by qPCR of the indicated cell lines (hPSC lines in grey bars and immortalised cell lines in orange bars). Bars indicate the mean of independent experiments shown as dots (n=3 for H9 and HaCat, for all the other cell lines n=2). Expression was normalised to the mean of H9 samples. Note that ZNF398 is expressed at levels comparable to hPSCs only in HaCaT and HEK293, while KLF4 is highly expressed in multiple cell lines. Source data are provided as a Source Data file. **b** Gene expression analysis by qPCR of HaCaT and HEK293 transfected with the indicated siRNA (siCONTROL, untransfected, siGFP, siZNF398 4 and siZNF398 5) and treated with SB43 (10 μM) for 16h (orange bars), followed by TGF-beta induction (5 ng/ml) for 6 hours (red bars, see Methods for additional details). Bars indicate the mean of independent experiments, shown as dots (n=2 for all conditions). Expression was normalised to the mean of siCONTROL SB43 samples. Source data are provided as a Source Data file. **c** Gene expression analysis by qPCR of HEK293 transiently expressing an empty vector or ZNF398 treated with SB43 (orange bars) or with TGF-beta induction (5 ng/ml) for 6 hours (red bars, see Methods for additional details). Bars indicate the mean of two independent experiments, shown as dots. Expression was normalised to the mean of Empty SB43 samples. Source data are provided as a Source Data file. **d** Gene expression analysis by qPCR of GOF18 and OEC2 (light and dark blue bars respectively) stably expressing an empty vector or Zfp398 treated with DMSO, SB43 (1 μM) or A83 (1μM) for 2 days. Bars indicate the mean of independent experiments shown as dots (n=1 in GOF18 and n=2 in OEC2). Expression was normalised to the Empty DMSO samples. Source data are provided as a Source Data file.

Supplementary Fig. 8

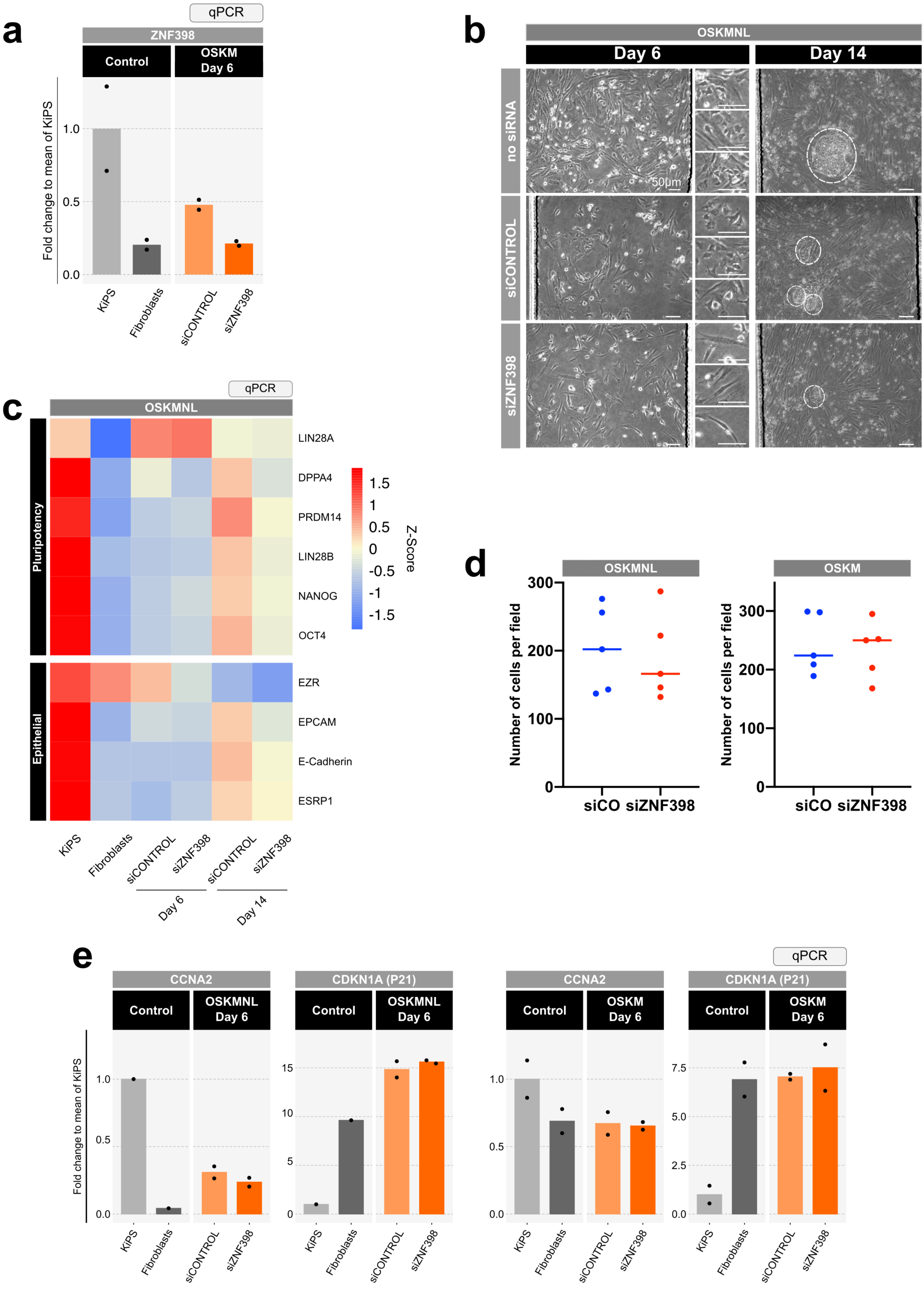

Supplementary Fig. 8: Somatic cell reprogramming by delivery of OSKM or OSKMNL mRNAs in combination with siRNA.

a Gene expression analysis by qPCR of hiPSCs (KiPS) (light grey) and BJ fibroblasts (dark grey) serving as controls, and BJ fibroblasts transfected with OSKM mRNAs and siCONTROL (light orange) or siZNF398 (dark orange) at day 6 of the reprogramming protocol described in Figure 5a. Bars indicate the mean of two independent experiments shown as dots. Expression was normalised to the mean of KiPS samples. See also Fig. 8e. Source data are provided as a Source Data file. **b** Cell morphology during OSKMNL mRNAs reprogramming. Representative images of four independent experiments are shown. See also Fig. 8b. **c** Heatmap showing gene expression analysis by qPCR for markers of pluripotency and epithelial character during reprogramming experiments. Z-Scores of expression values from 2 independent experiments are shown. Red and blue indicate high and low expression, respectively. See also Fig. 8e. Source data are provided as a Source Data file. **d** Bar plots showing the number of fibroblasts transfected with OSKM mRNAs and siCONTROL or siZNF398 at day 6 in OSKMNL and OSKM reprogramming respectively. For each condition, we took representative fields from 5 randomly selected biological replicates, like those shown in Supplementary Figure 8b. Each dot shows the number of cells per field. Bars indicate the mean of five biological replicates from a representative experiment. Source data are provided as a Source Data file. **e** Gene expression analysis by qPCR of hiPSCs (KiPS) (light grey) and fibroblasts (dark grey) serving as controls, and fibroblasts transfected with siCONTROL (light orange) or siZNF398 (dark orange) at day 6 in OSKMNL and OSKM reprogramming respectively. Bars indicate the mean of two independent experiments shown as dots. Expression was normalised to the mean of KiPS samples. Source data are provided as a Source Data file.

Supplementary Table 1: siRNA sequences information

Name	Product code	Sequence	Company
siCONTROL	SI03650318	UUCUCCGAACGUGUCACGU	Qiagen
siGFP	Custom	GCAAGCTGACCCTGAAGTTCA	Sigma
siNANOG 1	SI04341400	CACCCAATCCTGGAACAATCA	Qiagen
siNANOG 2	SI00654738	TTGGTTTAAGTTCAAATGAAT	Qiagen
siNANOG 3	SI05785122	AGGCCTTAATGTAATACAGCA	Qiagen
siNANOG 4	SI05785108	CTGCATGCAGTTCCAGCCAAA	Qiagen
siNANOG 5	SI05785115	CTCCATGGATCTGCTTATTCA	Qiagen
siZNF398 1	Custom	GAAGGGCAACTACGAGTCTCT	Sigma
siZNF398 2	SI05785164	CCGCAAGCACCACCTAATGAA	Qiagen
siZNF398 3	SI05785157	CAGGTGCGATACTAAGCCTCA	Qiagen
siZNF398 4	SI05785178	CTCCATGGATTATGCTATAAA	Qiagen
siZNF398 5	Custom	GAATCTCAGCCAAGACATGTT	Sigma

Supplementary Table 2: Antibodies details

Antibody	Dilution	Product code	Company	Antibody validation	Application
NANOG	1:100	D73G4	Cell Signaling TECHNOLOGY	Staining colocalises with nucleus in hPSCs in presence of TGF-beta and decrease upon TGF-beta deprivation. NANOG antibody was also validated by the provider: signal is present in NTERA-2 cells and absence in HeLa cells.	IF
POU5F1	1:300	sc-5279	Santa Cruz	Staining colocalizes with nucleus in hPSCs in presence of TGF-beta and decrease upon TGF-beta deprivation.	IF
VIMENTIN	1:100	sc-7557	Santa Cruz	Staining colocalises with cytoskeleton in hPSCs treated with SB43 and the signal is decreased in hPSCs cultured in the presence of TGF-beta.	IF
E-Cadherin	1:100	610181	BD	Staining colocalises with cytoskeleton in hPSCs cultered in the presence of TGF-beta and the signal is decreased in hPSCs treated with SB43.	IF
SMAD2/3	1:50	610843	BD	Staining colocalizes with cytoplasm in absence of TGF-beta and it shifts in the nucleus upon TGF-beta stimulation.	IF
phosho-SMAD3	1:100	52903	Abcam	Staining colocalises with the nucleus upon TGF-beta stimulation. Phosho-SMAD3 antibody was also validated by the provider using A549 cells untreated or treated with TGF-beta (5ng/ml, 24 h).	IF
phospho-SMAD1/5/8	1:100	AB3848	Millipore	Staining colocalizes with the nucleus upon BMP4 stimulation.	IF
Alexa Fluor 488 Phalloidin	1:200	A12379	ThermoFisher	Staining detects filamentous cytoplasmic structures that are lost upon treatment of cells with F-actin inhibitory drugs (Pocaterra et al. 2019 Journal of Hepatology).	IF

Donkey anti-Rabbit IgG (H+L) Secondary Antibody Alexa Fluor 488	1:500	A-21206	ThermoFisher	Internally validated	IF
Goat anti-Rabbit IgG (H+L) Secondary Antibody Alexa Fluor 568	1:500	A-11036	ThermoFisher	Internally validated	IF
Donkey anti-Mouse IgG (H+L) Secondary Antibody Alexa Fluor 488	1:500	A-21202	ThermoFisher	Internally validated	IF
Donkey anti-Mouse IgG (H+L) Secondary Antibody Alexa Fluor 647	1:500	A-31571	ThermoFisher	Internally validated	IF
Donkey anti-Mouse IgG (H+L) Secondary Antibody Alexa Fluor 568	1:500	A-10037	ThermoFisher	Internally validated	IF
SMAD3	2 µg	Ab28379	Abcam	SMAD3 antibody was previously validated in Mullen et al. Cell 2011. SMAD3 antibody was also internally validated in our experiments of ChIP-qPCR.	Co-IP
SMAD2	2 µg	D43B4	Cell Signaling TECHNOLOGY	SMAD2 antibody was validated by the provider in HaCat cells treated with TGF-beta for 1 hour and then immunoprecipated with SMAD2 or with a normal Rabbit IgG. Only in the enriched DNA immunoprecipated with SMAD2,	Co-IP

				two targets of TGF-beta (CDKN1A and ID1) were detected. SMAD2 antibody was also internally validated in our experiments of ChiP-qPCR.	
Avi-Tag	2 µg	A00674	GenScript	Avi-tag was validated by the provider using different amount of E.coli cell lysate an it was internally validated in our experiments of ChiP-qPCR.	Co-IP
Rabbit IgG	2 µg	12-370	Sigma-Aldrich	Rabbit IgG was validated by the provider and it is routinely evaluated as a non-specific IgG control.	Co-IP
phosho-Smad1/5/8	1:500	9511	Cell Signaling TECHNOLOGY	Phosho-Smad1/5/8 was validated by the provider in MEF cells untreated or treated with BMP2 and it was validated also in our lab with hPSCs culture for 16 hour in the presence of LDN 193189 and then untreated or treated with BMP4.	WB
phosho-Smad3	1:500	Ab52903	Abcam	Phosho-smad3 antibody was validated by the provider in A549 cell line untreated and treated with 5ng/ml TGF-beta for 24 hours and it was validated also in our lab with hPSCs cultured for 16 hour in the presence of SB431542 and then untreated or treated with TGF-beta.	WB
SMAD2/3	1:500	610843	BD	SMAD2/3 antibody was validated by the provider in Jurkat cells using different amount of lysate.	WB
GAPDH	1:25000	MAB374	Millipore	GAPDH antibody was recommended by the provider.	WB

Supplementary Table 3: qPCR primers details

Human Gene	Forward	Reverse
3'UTR	GTAATCATGGTCATAGCTGTTTCCT	CCAGGCTTTACACTTTATGCTTC
AFF4	TCTCAGTCTCAGAAACGGTCC	GGCTACTGCTCCCACTATTGTT
BCOR	CTCAGGGGCCACGAAACT	ATGGTGACAATTTCCACGTTT
CCRN4L	TCTCGCCAAGACACTGAACAG	GGCCCTGCATTCCTCAAGAAG
CDKN1A (p21)	CAGGAGGCCCGTGAGCGATGGA	ATCAGCCGGCGTTTGGAGTGGT
CXXC5	GCTGCTCTGGAGAAGGTGAT	GACCACACGAGCAGTGACAT
CCNA2	TTTGATAGATGCTGACCCATACC	ATGCTGTGGTGCTTTGAGGT
DPPA4	TCTGGTGTCAGGTGGTGTGT	TCCCTTCTTGCTTTTCTGGA
E-Cadherin	GGTCTGTCATGGAAGGTGCT	TCAGGATCTTGGCTGAGGAT
ENC1	TTGGAGCTGCTGCTTGACTA	TCCCGGATGTCTTGAAACTC
EPCAM	TGGACATAGCTGATGTGGCTTA	CCAGGATCCAGATCCAGTTG
ESRP1	CTTCCAACCCCTCCCATTAT	ATTGTGGCTGCATAGGGAAG
ETS2	CTGGGCATTCCAAAGAACCC	CCAGACTGAACTCATTGGTGG
ETV1	GGCCCCAGGCAGTTTTATGAT	GATCCTCGCCGTTGGTATGT
EZR	TGCAATCCAGCCAAATACAA	TTATCCAGCTTCAGCCAGGT
FLK1/KDR	GGCGGCACGAAATATCCTCT	GGAGGCGAGCATCTCCTTTT
FOXO1	TGATAACTGGAGTACATTTCGCC	CGGTCATAATGGGTGAGAGTCT
GAPDH	CGAGATCCCTCCAAAATCAA	GGCAGAGATGATGACCCTTT
GATA6	GCAAAAATACTTCCCCCACA	TCTCCCGCACCAGTCATC
GBX2	GTTCCACTGCAAAAAGTACCTCT	GGGACGACGATCTTAGGGTTC
ID1	ATCGCATCTTGTGTCGCTGA	GCCGATCGGTCTTGTTCTCC
JUNB	GACCAAGAGCGCATCAAAGT	CGTCTTCACCTTGTCCTCCA
KLF10	GCCAACCATGCTCAACTTCG	TGCAGTTTTGTTCCAGGAATACAT
KLF7	CTCATGGGAGGGATGTGAGT	ACCTGGAAAAACACCTGTCG
LEFTY1	GGACCTTGGGGACTATGGAG	ATCCCCTGCAGGTCAATGTA
LIN28A	CTGTAAGTGGTTCAACGTGCG	CCATGTGCAGCTTACTCTGGT
LIN28B	CCTCCTCAGCCAAAGAAGTG	TGGGATTCTGCTTCCTGTCT
MDF1	CTGGAGATCTGCATGGAGTG	AGAGGGACCCTGCTGGAAT
MYC	AGCGACTCTGAGGAGGAACA	GCTGTGAGGAGGTTTGCTGT
NANOG	TTTGTGGGCCTGAAGAAAACT	AGGGCTGTCCTGAATAAGCAG
N-Cadherin	CGTGGTCAAACCAATCGAC	AACAGACACGGTTGCAGTTG
NNAT	CCCACCATGGAAATCAAAAC	ACCACCCTCCTTCCTCAACT
OCT4	GTGGAGGAAGCTGACAACAA	ATTCTCCAGGTTGCCTCTCA
OTX2	CAAAGTGAGACCTGCCAAAAAGA	TGGACAAGGGATCTGACAGTG
PAX3	TCCACAAGCTGTGTCAGATCC	GCGTTGGAAGGAATCGTGCT
PAX6	TGGGCAGGTATTACGAGACTG	ACTCCCGCTTATACTGGGCTA
PRDM14	GAGCCTTCAGGTCACAGAGC	TCCACACAGGGGGTGTACTT
S100A4	AGCTTCTTGGGGAAAAGGAC	TCTTGGAAGTCCACCTCGTT
SIP1	CGCTTGACATCACTGAAGGA	CTTGCCACACTCTGTGCATT
SIX3	CCCCACCTGCATGACGATTT	TCTCCTCGTGGTGGTGATTG
SKIL	TATGCAGGACAGTTGGCAGA	TTCTGTCTTGCTTCCCGTTC
SMAD7	CAAGAGGCTGTGTTGCTGTG	GGGAGACTCTAGTTCGCAGA

SOX17	ACGCCGAGTTGAGCAAGA	TCTGCCTCCTCCACGAAG
T	TATGAGCCTCGAATCCACATAGT	CCTCGTTCTGATAAGCAGTCAC
TBL1XR1	GGCAGAGCAACAACACCTTT	TGGGTCCCATTTGATAGCAT
TEAD1	ATGCCAACCATTCTTACAGTGAC	ACAGTTCCTTTAAGCCACCTTTC
TLE3	TTGAGCCGATACGACAGTGA	CTTGTCCAGCCCATTTTCAG
UTF1	CTCCCAGCGAACCAGACG	GGAGGCGTCCGCAGACTT
VIM	CTCCACGAAGAGGAAATCCA	GTGAGGTCAGGCTTGGAAAC
ZFP42	GCCTTATGTGATGGCTATGTGT	ACCCCTTATGACGCATTCTATGT
ZNF398	TGGCAAGAATCTCAGCCAAGA	GTGGAGTAAAGTGCTTAGGGC

Mouse gene	Forward	Reverse
Fgf5	gcgacgttttcttcgtcttc	gatgcccactctgcagtaca
Lefty1	gttcagccagaaccttcgag	gctccattccgaacactagc
Lefty2	aatggctgagagtccgtgag	ggatggacacgagcctagag
Nanog	ttcttgcttacaagggtctgc	agaggaagggcgaggaga
Nodal	ctggcgtacatgttgagcct	ggtcacgtccacatcttgcg
Oct4	gttggagaaggtggaaccaa	ctccttctgcagggctttc
Otx2	ggaagaggtggcactcaaaa	ggcctcactttgttctgacc
Smad7	cccctcctccttactccaga	caggctccagaagaagttgg
Zfp398	ggtacctatgctgatgaagaacttg	ctggtggtggagcagagaa

Supplementary Table 4: ChIP-qPCR primers details

Gene	Forward	Reverse
ESRP1	CCGCCAGGTGTCTCTTACC	TTGGGATGTGGCTTTACCTGC
LEFTY1	GAAGCTCACAGCCAGACGA	CCCTATCTAGACAGCCCCCTC
LIN28B	CAGTGGGGCTTTCTGCAAAC	GAATCAGTGTGGGCGGTACA
NANOG	ATGGGCTTTGACTCAAGGGG	GTGCCGGAAGCTTTTGTCTG
NEGATIVE 1	GTGGGTTGTTGCTGTTGTTCTA	ACTACACTCAAGGCAGCTGGA
NEGATIVE 2	CCCCGCCAGTATCATCTCTG	ACTCTGCAGACCGTCAGATG
SMAD7	TGTCTTCTGTGTGCCAGACTC	GCTCTTGTGGTGTGAACGTC
ZNF398	CAACCCAACTCCCAGGTACA	GAGTCCCAGCATGCACCG

Supplementary References

1. Rada-Iglesias, A. *et al.* A unique chromatin signature uncovers early developmental enhancers in humans. *Nature* **470**, 279–283 (2011).
2. Bailey, T. L. *et al.* MEME SUITE: tools for motif discovery and searching. *Nucleic Acids Res.* **37**, W202-208 (2009).
3. Uhlén, M. *et al.* Tissue-based map of the human proteome. *Science* **347**, (2015).
