# Supplementary material for: The transcriptional regulator ZNF398 mediates pluripotency and epithelial character downstream of TGF-beta in human PSCs
Source: Nat Commun. 2020 May 12;11:2364. doi: 10.1038/s41467-020-16205-9 (PMC7217929; doi:10.1038/s41467-020-16205-9)
Supplement: Supplementary file 5 — Description of Additional Supplementary Files [file 41467_2020_16205_MOESM5_ESM.pdf]

Title: Supplemental Data 1:
Description: Microarray and RNA-seq data used for TGF-beta/SMAD3 targets identification.

Title: Supplemental Data 2:
Description: Differential expression analysis of KiPS transfected with the empty vector and treated with SB43 for 5 days and absolute expression (TPM) of KiPS stably transfected with the empty vector, NANOG, KLF7, MYC or ZNF398 in presence of DMSO or SB43 for 5 days.
