# Supplementary material for: The transcriptional regulator ZNF398 mediates pluripotency and epithelial character downstream of TGF-beta in human PSCs
Source: Nat Commun. 2020 May 12;11:2364. doi: 10.1038/s41467-020-16205-9 (PMC7217929; doi:10.1038/s41467-020-16205-9)
Supplement: Supplementary file 6 — Reporting Summary [file 41467_2020_16205_MOESM6_ESM.pdf]

nature research

Corresponding author(s): Salvatore Oliviero
Graziano Martello

Last updated by author(s): Apr 8, 2020

Reporting Summary

Nature Research wishes to improve the reproducibility of the work that we publish. This form provides structure for consistency and transparency in reporting. For further information on Nature Research policies, see Authors & Referees and the Editorial Policy Checklist .

Statistics

For all statistical analyses, confirm that the following items are present in the figure legend, table legend, main text, or Methods section.

n/a	Confirmed	
☐	☒	The exact sample size (*n*) for each experimental group/condition, given as a discrete number and unit of measurement
☐	☒	A statement on whether measurements were taken from distinct samples or whether the same sample was measured repeatedly
☐	☒	The statistical test(s) used AND whether they are one- or two-sided<br>*Only common tests should be described solely by name; describe more complex techniques in the Methods section.*
☐	☒	A description of all covariates tested
☐	☒	A description of any assumptions or corrections, such as tests of normality and adjustment for multiple comparisons
☐	☒	A full description of the statistical parameters including central tendency (e.g. means) or other basic estimates (e.g. regression coefficient) AND variation (e.g. standard deviation) or associated estimates of uncertainty (e.g. confidence intervals)
☐	☒	For null hypothesis testing, the test statistic (e.g. *F*, *t*, *r*) with confidence intervals, effect sizes, degrees of freedom and *P* value noted<br>*Give P values as exact values whenever suitable.*
☒	☐	For Bayesian analysis, information on the choice of priors and Markov chain Monte Carlo settings
☒	☐	For hierarchical and complex designs, identification of the appropriate level for tests and full reporting of outcomes
☒	☐	Estimates of effect sizes (e.g. Cohen's *d*, Pearson's *r*), indicating how they were calculated

Our web collection on statistics for biologists contains articles on many of the points above.

Software and code

Policy information about availability of computer code

Data collection	qPCR data were acquired with QuantStudio™ 6&7 Flex Software 1.0. Immunostaining data were acquired with Leica TCS SP5 LAS AF (v2.7.3.9723) and ZEN 2012 softwares. Western blotting was acquired with LAS400 ImageQuant 1.2.

Data analysis

R software (v3.5.2) was used for all statistical analysis.
Fiji 1.0 (Imaje2) and CellProfiler (v3.1.8) were used for images analysis.
For published microarray data (E-MEXP-1741), differential expression analysis was performed using limma (v3.18.13) on the RMA normalized gene expression matrix.
For RNA-seq of induction experiments, raw reads were aligned in two steps: first reads were aligned on genome build GRCh37.p13 with STAR (v2.4), reads that were not aligned in this step were realigned with bowtie2 (v2.2.4). Raw counts over the ensembl annotation release 75 were obtained with htseq-count (v 0.6.0). Normalization of raw reads and differential analysis were carried out using edgeR package (v3.4.2) and R (v3.5.2).
For RNA-seq of overexpression experiments, reads were mapped to the Homo sapiens hg19 reference assembly using TopHat (v2.1.1), and gene counts were computed using htseq-count (v0.6.1p1). Differential expression analysis was performed using DESeq v2.
For ChIP-seq data of SMAD3 in BG03 cell line, raw reads were aligned using Bowtie (v0.12.7) to build version hg19 of the human genome retaining only uniquely mapped reads. Redundant reads were removed using SAMtools (v0.1.18). MACS2 (v2.0.10)was used to call peaks for SMAD3 using WCE ChIP-seq as control sample and setting the bandwidth equal to the estimated sonication fragment size (131 bp) and the p-value cutoff at 0.01. Only peaks with a pileup height greater than 5 were kept for further analysis. Each peak was assigned to the nearest TSS in a window of 100 Kb centered on the peak, considering only protein coding genes in GENCODE v16 annotation.
For ChIP-seq data of ZNF398 in H9 and BG01V lines, reads were mapped to the Homo sapiens hg19 reference assembly using Bowtie (v1.2.2), keeping only uniquely mapped reads. Reads (75 bp) were bioinformatically extended to the average insert size (150 bp), and identical reads (reads starting and ending at the same positions) were collapsed. Peak calling was performed using MACS (v2.1.1), selecting only peaks with q-value < 0.05. A non-redundant set of common peaks between the two ZNF398 ChIP-seq replicates was generated using the intersectBed utility from BEDTools (v2.26.0). For motif discovery, peaks were resized to ±200 bp surrounding their center and motif discovery was performed using MEME (v4.10.1).

For manuscripts utilizing custom algorithms or software that are central to the research but not yet described in published literature, software must be made available to editors/reviewers. We strongly encourage code deposition in a community repository (e.g. GitHub). See the Nature Research guidelines for submitting code & software for further information.

Data

Policy information about availability of data

All manuscripts must include a data availability statement. This statement should provide the following information, where applicable:

- Accession codes, unique identifiers, or web links for publicly available datasets
- A list of figures that have associated raw data
- A description of any restrictions on data availability

RNA-seq and ChIP-seq data of this study have been deposited on Gene Expression Omnibus (GEO) database, under the accession GSE133630.
For the identification of TGF-beta transcriptional targets, we used available SMAD3 ChIP-seq data (Accession Number GSE21621), microarray data (Accession Number E-MEXP-1741) and RNA-seq data of H9, (Accession Number GSE24447, see Supplementary Fig. 1e).
For correlation analyses and comparison of ZNF398 genome occupancy with known factors/histone modifications, data was collected from the GEO database for the following datasets: GSE54471 (H3K27ac and H3K4me1), GSE76084 (H3K27me3, H3K36me3, H3K4me3, H3K9ac, SOX2), GSE118325 (H3K9me3), GSE73725 (NANOG). Data for POU5F1 and EP300 was instead obtained from the ENCODE database (https://www.encodeproject.org/).
All plasmids, materials and data supporting the findings of this study will be available from corresponding authors upon reasonable request. The source data underlying Figs 1b, 2a, b, 3a, c, 4a, 5d, e, 6c, 7a, b, 8c, e and Supplementary Figs 1b, f, g, 2d, 3a, c, d, 4a, b, 5a-c, 6b, 7a-d, 8a, c-e are provided as a Source Data file.

Field-specific reporting

Please select the one below that is the best fit for your research. If you are not sure, read the appropriate sections before making your selection.

☒ Life sciences ☐ Behavioural & social sciences ☐ Ecological, evolutionary & environmental sciences

For a reference copy of the document with all sections, see nature.com/documents/nr-reporting-summary-flat.pdf

Life sciences study design

All studies must disclose on these points even when the disclosure is negative.

Sample size	Sample size calculation was not performed. Sample size was chosen based on standards in the field (Xu et al. 2008 Cell Stem Cell; Vallier et al. 2009 Development; Chia et al. 2010 Nature; Wang et al. 2012 Cell Stem Cell) and is indicated in figure legends. All key experiments were repeated independently using different cell lines.
Data exclusions	We presented all data obtained, also in the case of negative results.
Replication	All key experiments were repeated between 2 and 7 times independently, as indicated. Experiments of candidates functional validation were repeated using 3 different hPSC lines. All qPCR experiments were performed with three technical replicates. All attempts of replication were successful.
Randomization	Randomization was not relevant. All cell lines or biological samples were analysed or treated in the same manner.

Blinding	We applied blinding for RNA-seq and ChIP-seq processing and data analysis. All other analysis were not blinded because this was not applicable, but key data were independently replicated by two different operators.

Reporting for specific materials, systems and methods

We require information from authors about some types of materials, experimental systems and methods used in many studies. Here, indicate whether each material, system or method listed is relevant to your study. If you are not sure if a list item applies to your research, read the appropriate section before selecting a response.

Materials & experimental systems

n/a	Involved in the study	
☐	☒	Antibodies
☐	☒	Eukaryotic cell lines
☒	☐	Palaeontology
☒	☐	Animals and other organisms
☒	☐	Human research participants
☒	☐	Clinical data

Methods

n/a	Involved in the study	
☐	☒	ChIP-seq
☒	☐	Flow cytometry
☒	☐	MRI-based neuroimaging

Antibodies

Antibodies used

Antibodies for immunofluorescence: NANOG (1:100, Cell Signaling TECHNOLOGY, D73G4), POU5F1 (1:300, Santa Cruzs, c-5279), VIMENTIN (1:100, Santa Cruz, sc-7557), E-Cadherin (1:100, BD, 610181), SMAD2/3 (1:50, BD, 610843), phosho-SMAD3 (1:100, Abcam, 52903), phospho-SMAD1/5/8 (1:100, Millipore, AB3848), Alexa Fluor 488 Phalloidin (1:200, ThermoFisher, A12379), Donkey anti-Rabbit IgG (H+L), Secondary Antibody Alexa Fluor 488 (1:500, ThermoFisher, A-21206), Goat anti-Rabbit IgG (H+L) Secondary Antibody Alexa Fluor 568 (1:500, ThermoFisher, A-11036), Donkey anti-Mouse IgG (H+L) Secondary Antibody Alexa Fluor 488 (1:500, ThermoFisher, A-21202), Donkey anti-Mouse IgG (H+L) Secondary Antibody Alexa Fluor 647 (1:500, ThermoFisher, A-31571), Donkey anti-Mouse IgG (H+L) Secondary Antibody Alexa Fluor 568 (1:500, ThermoFisher, A-10037).

Antibodies for Coimmunoprecepitation: SMAD3 (2 µg, Abcam, Ab28379), SMAD2 (2 µg, Cell Signaling TECHNOLOGY, D43B4), Avi-tag (2 µg, GenScript, A00674), Rabbit IgG (2 µg Sigma-Aldrich, 12-370).

Antibodies for Western blotting: phosho-Smad1/5/8 (1:500, Cell Signaling TECHNOLOGY, 9511), phosho-Smad3 (1:500, Abcam, Ab52903), SMAD2/3 (1:500, BD, 610843), GAPDH (1:25000, Millipore, MAB374).

Validation

Antibodies used for immunofluorescence:

NANOG staining colocalises with nucleus in hPSCs in presence of TGF-beta and decrease upon TGF-beta deprivation. NANOG antibody was also validated by the provider: signal is present in NTERA-2 cells and absence in HeLa cells.

POU5F1 staining colocaliseswith nucleus in hPSCs in presence of TGF-beta and decrease upon TGF-beta deprivation.

VIMENTIN staining colocalises with cytoskeleton in hPSCs treated with SB43 and the signal is decreased in hPSCs cultured in the presence of TGF-beta.

E-Cadherin staining colocalises with cytoskeleton in hPSCs cultered in the presence of TGF-beta and the signal is decreased in hPSCs treated with SB43.

SMAD2/3 staining colocalises with cytoplasm in absence of TGF-beta and it shifts in the nucleus upon TGF-beta stimulation.

Phosho-SMAD3 staining colocalises with the nucleus upon TGF-beta stimulation. Phosho-SMAD3 antibody was also validated by the provider using A549 cells untreated or treated with TGF-beta (5ng/ml, 24 h).

Phospho-SMAD1/5/8 staining colocalises with the nucleus upon BMP4 stimulation.

Alexa Fluor 488 Phalloidin staining detects filamentous cytoplasmic structures that are lost upon treatment of cells with F-actin inhibitory drugs (Pocaterra et al. 2019 Journal of Hepatology).

Antibodies used for coimmunoprecipitation:

SMAD3 was previously validated in Mullen et al. Cell 2011. SMAD3 antibody was also internally validated in our experiments of ChIP-qPCR.

SMAD2 was validated by the provider in HaCat cells treated with TGF-beta for 1 hour and then immunoprecipated with SMAD2 or with a normal Rabbit IgG. Only in the enriched DNA immunoprecipated with SMAD2, two targets of TGF-beta (CDKN1A and ID1) were detected. SMAD2 antibody was also internally validated in our experiments of ChiP-qPCR.

Avi-tag was validated by the provider using different amount of E.coli cell lysate an it was internally validated in our experiments of ChiP-qPCR.

Rabbit IgG was validated by the provider and it is routinely evaluated as a non-specific IgG control.

Antibodies used for Western blotting:

Phosho-Smad1/5/8 was validated by the provider in MEF cells untreated or treated with BMP2 and it was validated also in our lab with hPSCs culture for 16 hour in the presence of LDN 193189 and then untreated or treated with BMP4.

Phosho-Smad3 was validated by the provider in A549 cell line untreated and treated with 5ng/ml TGF-beta for 24 hours and it was validated also in our lab with hPSCs cultured for 16 hour in the presence of SB431542 and then untreated or treated with TGF-beta.

SMAD2/3 was validated by the provider in Jurkat cells using different amount of lysate.

GAPDH was recommended by the provider.

Eukaryotic cell lines

Policy information about cell lines

Cell line source(s)	H9 (WA09) cell line was obtained from WiCell.<br>KiPS (Keratinocytes induced Pluripotent stem cell) and OEC2 were provided by Austin Smith's laboratory.<br>GOF18 were provided by Hans R. Schöler's laboratory.<br>HES2 cell line was provided by Nicola Elvassore's laboratory.<br>MEF DR4 and BJ were obtained from ATCC.<br>BG01V/hOG (BG01V) were obtained from Fisher Scientific (Gibco R7799105).<br>HaCaT cell line was kindly provided by Stefano Piccolo's laboratory.<br>HEK293T, A549, MCF10AneoT, MCF10A, MCF10CA1a, HepG2, MDA-MB-231, WI-38 and RPE1 were kindly provided by Sirio Dupont's laboratory.
Authentication	H9 cell line was authenticated by STR profiling by WiCell.<br>MEF DR4, BJ, HEK293T, A549, MDA-MB-231 were authenticated by STR profiling by ATCC.<br>WI-38 were authenticated by STR profiling by ECACC.<br>HES2 were authenticated by STR profiling by WiCell.<br>BG01V/hOG cells were derived from the BG01V human embryonic stem cell (hESC) line (ATCC No. SCRC-2002) (Mitalipova et al. 2003; Plaia et al. 2006). BG01V cells stain positively for pluripotency markers and can differentiate to representatives of all three primary germ layers. An integration vector containing an EmGFP (Emerald Green Fluorescent Protein) gene directed by the human Oct4 promoter and followed by the HSV-TK polyadenylation signal was stably integrated into the genome of BG01V cells. The engineered cells express EmGFP under the appropriate conditions when the Oct4 promoter is active. This promoter is only active when hESCs are in the pluripotent state and therefore acts as a sensitive indicator of differentiation (Pan et al. 2002).<br>KiPS were generated in Austin Smith's laboratory (Takashima et al. Cell Stem Cell 2014) by somatic cell reprogramming with Sendai virus (OSKM). KiPS were not authenticated. In our lab, we confirmed that KiPS line maintain pluripotency markers and clonogenic ability unless challenged with differentiating condition.<br>GOF18 were derived in Hans R. Schöler's laboratory (Han et al. Cell 2010). GOF18 were not authenticated. In our lab, we confirmed that GOF18 line maintain pluripotency markers and clonogenic ability unless challenged with differentiating condition.<br>OEC2 were derived in Austin Smith's laboratory (Yang et al. Cell Stem Cell 2010). OEC2 were not authenticated. In our lab, we confirmed that OEC2 line maintain pluripotency markers and clonogenic ability unless challenged with differentiating condition.<br>MCF10AneoT were derived by S.J. Santer (S.J.Santer et al. Breast Cancer Research and treatment 2001) and they were authenticated by PCR in the mutated gene MAP3K12 by Sirio Dupont's laboratory.<br>HepG2, MCF10A (S.J.Santer et al. Breast Cancer Research and treatment 2001), MCF10CA1a (S.J.Santer et al. Breast Cancer Research and treatment 2001), RPE-1 and HaCaT were not authenticated.
Mycoplasma contamination	Cells were routinely tested for Mycoplasma contamination. All cell lines were Mycoplasma negative.
Commonly misidentified lines<br>(See ICLAC register)	The cell lines used are not listed in ICLAC.

ChIP-seq

Data deposition

- [x] Confirm that both raw and final processed data have been deposited in a public database such as GEO.
- [x] Confirm that you have deposited or provided access to graph files (e.g. BED files) for the called peaks.

Data access links<br>*May remain private before publication.*	To review GEO accession GSE133630:<br>Go to https://www.ncbi.nlm.nih.gov/geo/query/acc.cgi?acc=GSE133630<br>Enter token ghepogsgbrwdzar into the box
Files in database submission	ChIP-seq ZNF398 in H9, ChIP-seq ZNF398 in BG01V
Genome browser session<br>(e.g. UCSC)	no longer applicable

Methodology

Replicates	2 biological replicates
Sequencing depth	single read, 75bp<br># of unique mappings (following identical reads collapsing): 2356447 (H9), 9433452 (BG01V)

Antibodies	ZNF398 bearing Avitag at N-Term was co-expressed with birA enzyme (E. coli) in H9 and BG01V cells. Immunoprecipitation was performed using streptavidin beads.
Peak calling parameters	Peaks were called using macs2, with minimum FDR set to 0.05.
Data quality	All peaks have FDR <= 5%. >50% peaks have fold-enrichment >= 5.
Software	Mapping was performed using Bowtie v1.2.1, allowing only unique mappings (--best--strata -m 1). Peak calling was performed using macs 2.1.1, with FDR cutoff at 0.05 (-q 0.05).
